# Engineering monoclonal antibody-based contraception and multipurpose prevention technologies^†^

**DOI:** 10.1093/biolre/ioaa096

**Published:** 2020-06-08

**Authors:** Deborah J Anderson, Joseph A Politch, Richard A Cone, Larry Zeitlin, Samuel K Lai, Philip J Santangelo, Thomas R Moench, Kevin J Whaley

**Affiliations:** 1 Department of Medicine, Boston University School of Medicine, Boston, MA, USA; 2 Biophysics Department, Johns Hopkins University, Baltimore, MD, USA; 3 Mucommune, LLC, Durham, NC, USA; 4 ZabBio, Inc., San Diego, CA, USA; 5 Division of Pharmacoengineering and Molecular Pharmaceutics, Department of Microbiomology & Immunology, University of North Carolina, Chapel Hill, NC, USA; 6 Wallace H. Coulter Department of Biomedical Engineering, Georgia Institute of Technology and Emory University Atlanta, GA, USA

**Keywords:** Sperm, monoclonal antibody, multipurpose prevention technology, contraception, sexually transmitted infections

## Abstract

Sexually transmitted infections are highly prevalent, and over 40% of pregnancies are unplanned. We are producing new antibody-based multipurpose prevention technology products to address these problems and fill an unmet need in female reproductive health. We used a *Nicotiana* platform to manufacture monoclonal antibodies against two prevalent sexually transmitted pathogens, HIV-1 and HSV-2, and incorporated them into a vaginal film (MB66) for preclinical and Phase 1 clinical testing. These tests are now complete and indicate that MB66 is effective and safe in women. We are now developing an antisperm monoclonal antibody to add contraceptive efficacy to this product. The antisperm antibody, H6-3C4, originally isolated by Shinzo Isojima from the blood of an infertile woman, recognizes a carbohydrate epitope on CD52g, a glycosylphosphatidylinositol-anchored glycoprotein found in abundance on the surface of human sperm. We engineered the antibody for production in *Nicotiana*; the new antibody which we call “human contraception antibody,” effectively agglutinates sperm at concentrations >10 μg/ml and maintains activity under a variety of physiological conditions. We are currently seeking regulatory approval for a Phase 1 clinical trial, which will include safety and “proof of principle” efficacy endpoints. Concurrently, we are working with new antibody production platforms to bring the costs down, innovative antibody designs that may produce more effective second-generation antibodies, and delivery systems to provide extended protection.

## Teaser

We are engineering monoclonal antibodies and delivery systems for multipurpose prevention technology products that will concurrently prevent sexually transmitted infections and unplanned pregnancies, addressing an important unmet need in reproductive health.

For over 10 years, our research team has been working on monoclonal antibody (mAb)-based multipurpose prevention technology (MPT) products to protect women against sexually transmitted infections (STIs) while at the same time providing contraception to prevent unplanned pregnancies. As STIs are highly prevalent [1], and over 40% of pregnancies worldwide are unplanned [2], MPTs could fill a critical gap. Recent surveys indicate that a large number of women around the world would choose to use an MPT product if available [3–5]. Since antibodies naturally occur in blood and genital secretions to provide protection [6], human antibody-based MPT products that fortify this natural barrier should pose low risk. Our first product, MB66, is a vaginal film that contains mAbs directed against two incurable sexually transmitted viruses, HIV-1 (VRC01) and herpes simplex viruses 1 and 2 (HSV8). Preclinical studies indicate that the antibodies in MB66 remain active under low pH genital tract conditions and effectively prevent macaques from vaginal SHIV transmission [7]. MB66 was recently tested in a Phase 1 clinical trial and single and multiple dose applications proved to be safe; furthermore, both the HIV and HSV antibodies retained neutralizing activity in vaginal secretions for at least 24 h after film application [8]. We now seek to add an antisperm mAb to MB66 to provide a contraception option for women. In this review article, we discuss: (1) naturally occurring and manufactured antibodies that target and reduce the fertility potential of human sperm; (2) various functions of antisperm antibodies that can affect fertility; (3) selection and manufacture of an antisperm mAb candidate (“human contraceptive antibody”—HCA); (4) engineering antisperm mAbs with enhanced function; and (5) contraceptive mAb delivery systems.

## Antisperm antibodies

**Figure 1:**
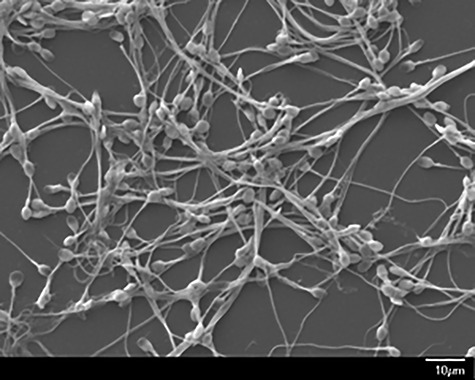
Scanning electron micrograph showing agglutination of HCA-treated washed human spermatozoa.

Throughout the 20th century, there was considerable interest in naturally occurring antisperm antibodies found in the blood and genital secretions of a sizeable percentage of men and women with unexplained infertility. Sperm can induce an immune reaction in men after vasectomy or other events that affect the patency of the male tract, and in some women that, for unknown reasons, are not tolerant to their partner’s sperm [9]. Antisperm antibodies are detected clinically by assays that assess sperm agglutination, complement-mediated sperm cytotoxicity, or binding of antibodies to the sperm surface (immunobead assay), and were a routine aspect of the assessment of infertile couples [10]. Antisperm antibodies were detected in as many as 15% of couples with unexplained infertility [11]. Many scientists surmised that antisperm antibodies caused infertility, and developed antisperm mAbs to use as tools to identify antigens on the sperm surface that could be targeted for immunocontraception [12] (Table 1). We selected an IgM antisperm mAb, H6-3C4, originally isolated by Isojima et al. [13] from blood cells of an infertile woman; the nucleotide sequence of the variable region of H6-3C4 was published by the group 1 year later [14]. This mAb is directed against a carbohydrate epitope on CD52g, a small glycoprotein found in abundance on the sperm surface [15], and is a very potent mediator of sperm cytotoxicity and agglutination [13] (Figure 1).

**Table 1:** Sperm-associated antigens that have been implicated in immunocontraception.

Name	Description	Reference
Izumo	Ig-like domain on sperm	[92–94]
LDH-C4	Sperm lactate dehydrogenase	[95]
PH-20	Hyaluronidase	[96]
FA-1	Zona pellucida binding protein	[97]
H6-3C4, SAGA-1	CD52g	[15]
YLP12	Zona pellucida binding protein	[98]
SAMP32	Oocyte binding protein	[99]
Eppin	Epididymal protease inhibitor	[100]

Since it is difficult to produce IgM antibodies in the Nicotiana system, we converted the isotype of the antibody to IgG. Genes containing the variable region sequences of H6-3C4 were cloned into TMV and PVX plant expression vectors [16] containing codon-optimized human lambda and human IgG_1_ constant regions. The vectors were then transformed into *Agrobacterium tumefaciens* strain ICF320 for transfection into *Nicotiana benthamiana* plants by vacuum infiltration as previously described [17]. After 7 days of post-infiltration, antibody was extracted from the leaf tissue and purified by Protein A chromatography [17, 18]. We refer to this IgG1 antisperm mAb as HCA.

## Functions of antisperm antibodies that can affect fertility

Antibodies achieve protective effects through a variety of mechanisms [7]: (1) Antibodies can directly block the function of targeted molecules (i.e., neutralizing pathogens by blocking critical receptors). Some antisperm mAbs may use this mechanism to block molecules that execute critical sperm functions such as the acrosome reaction or sperm–egg interactions [12, 19]. (2) Antibodies can interact with Fc receptors on immune cells to activate effector functions such as antibody-dependent phagocytosis and cellular cytotoxicity. However, there are usually few immune cells in the superficial layers of the genital tract epithelium and lumen, so these effector functions may not play a major role in sperm inactivation. (3) Antibodies can bind complement to effect complement-dependent cytotoxicity. This mechanism has been demonstrated for sperm antibodies when external complement sources (e.g., Guinea pig serum) are used, but may not be a major mechanism mediating sperm cytotoxicity in the genital tract because complement concentrations at this site are thought to be low [20].

Two “nonclassical” antibody mechanisms are thought to be critical for observed infertility, based on limiting sperm movement through genital tract mucus: agglutination and mucus trapping. Since sperm are motile, and are delivered at high concentrations in the ejaculate, antisperm antibodies can agglutinate sperm, crosslinking them into thick “cables” from which they rarely escape. In addition, antibodies can trap (immobilize) individual sperm in mucus gel by forming weak but polyvalent crosslinks between the sperm and the gel-forming mucin fibers. Castle et al. [21] developed the rabbit contraceptive model to test whether sperm-agglutinating monoclonal antibodies (mAbs) were effective contraceptives. By immunizing mice with rabbit sperm, they obtained three sperm-agglutinating mAbs, one IgM, and two IgGs. Rabbits were vaginally inseminated with ~2500 fertilizing doses of rabbit sperm mixed with various amounts of each mAb (in comparison the human ejaculate contains about one fertilizing dose). The most potent mAb, on a mass basis, was an IgG that was ~80% contraceptive at a dose of 100 ng/ml IgG. The IgM was about equally effective on a molar basis.

Mucus trapping is a little recognized, but potent, function that antibodies can perform in mucus gels. It was first reported by Kremer and Jager [22] who observed that individual sperm that had penetrated endocervical mucus in infertile women shook in place (“shaking phenomenon”). The antibodies did not kill the sperm, but simply crosslinked the sperm to the mucus gel, thereby preventing forward motility. They hypothesized that the antibodies that bound to the sperm were also strongly attached to the gel-forming mucin fibers [23].

Mucus is a viscid (sticky) gel that adheres to many types of surfaces, both hydrophilic and hydrophobic. Antibodies and mucins are both negatively charged and hence should repel each other. Interestingly, the diffusion of antibodies is somewhat impeded by mucus gels: IgG is slowed by only ~10% while IgM with its 5 Fc moieties, is slowed by ~50%. Such modestly slowed diffusion is consistent with antibodies making weak and transient bonds with the mucins. In turn, the weak affinity of individual antibody–mucin bonds allow individual antibodies to diffuse rapidly within the mucus gel, thereby enabling them to contact and coat the surfaces of pathogens or sperm to which they bind specifically [24, 25]. As the antibodies assemble on the surface of sperm (or pathogens), they form an array of Fc moieties that can make multiple low-affinity bonds to the mucus gel. Such an array can trap vigorously flagellating sperm, as well as highly motile bacteria such as *Salmonella typhimurium* in mouse intestinal mucus [26], as well as much smaller viruses such as Herpes [27], influenza [28], and Ebola [29]. In these cases, the antibodies trap the motile organisms without agglutinating them, or killing them. Long ago it was demonstrated that antisperm antibodies can trap vigorously motile sperm in human mid-cycle cervical mucus without killing them (the “shaking phenomenon”) [30] and that with sIgA the Fc region of the antibody mediated the trapping action [31]. Trapping can be extremely potent: the human antisperm IgM mAb first isolated by Isojima [13] can trap individual sperm in endocervical mid-cycle mucus at 1–10 ng/ml [32, 33], and vaginal Herpes can be trapped at subneutralizing IgG concentrations [27].

Trapping in mucus appears to effectively block pathogen contact with target cells; vaginal application of a non-neutralizing (but HSV-trapping) IgG blocked vaginal transmission of HSV in the mouse genital herpes model [27]. Deglycosylation or removing the Fc component from purified anti-HSV antibodies, markedly reduced trapping potency. These observations suggest that IgG-Fc has a glycan-dependent “muco-trapping” effector function that may provide exceptionally potent protection at mucosal surfaces.

## Manufacturing the human contraceptive antibody

There are over 100 mAbs approved or under review for clinical use in the US and EU [34]. A few approved antibodies are against infectious diseases (e.g., palivizumab for RSV), and there are ongoing efficacy trials of broadly neutralizing antibodies against HIV (ClinicalTrials.gov Identifier: NCT02568215). However, the HCA is the only antisperm antibody currently in advanced development. The attributes of HCA as a contraceptive only, or as a component of an MPT product are described in Table 2.

**Table 2:** Target product profile for HCA and MPT.

Variable	Minimum	Optimistic
Indication	Prevention of unwanted pregnancy	Prevention of HIV, HSV, and unwanted pregnancy
Product	Contraceptive only (HCA): sperm agglutination and mucus trapping	Contraceptive MPT: viral neutralization, mucus trapping, and sperm agglutination
Target population	Sexually active women	Sexually active girls and women
Target countries	Worldwide	Worldwide
Efficacy	Comparable to typical condom use	Comparable to perfect condom use
Safety	Slight irritation, minimal disruption of microbiome	No irritation and no disruption of microbiome
Duration of protection	2 h	24 h
Formulation dosage and route of administration	Vaginal film (on demand method) 10 mg/intercourse	Film and 30-day intravaginal ring (non-coital, long-acting method) 100–300 mg/month
Stability/shelf life	4 °C in stores for 2 years; 6 months ambient temperature	Ambient temperature for 2 years
Product registration path	Contraceptive claim only	Contraceptive claim followed by HIV and HSV claims
WHO prequalification date	2027	2027
Primary target delivery channel	Health care facility, public funding	Direct to consumer and health care facility, public and private funding
COGS	$0.30/film	$0.20/film; $3.00/ring

### Regulatory strategy

The HCA project leverages the scientific and regulatory knowledge generated by the CMC manufacturing, preclinical development, and Phase 1 clinical trial of the anti-viral mAb-based vaginal microbicide MB66 (IND #122,010). The HCA film will be evaluated with the standardized post-coital test (PCT) in a first in human mechanism of action and surrogate efficacy study. Although this test is no longer widely used to evaluate infertility, it is still used to assess the efficacy of vaginal contraceptive products [35–38]. If the planned Phase 1 “mechanism of action” clinical study is successful in showing that a vaginal film containing HCA (ZB-06) prevents progressively motile sperm from accessing the cervix after intercourse in tubally ligated women, it will provide an essential demonstration of mechanism of action for subsequent contraceptive product development. The next step after this exploratory IND study will be to focus on a new combination product containing both contraceptive and anti-viral mAbs, with the intent to carry it through to efficacy testing and commercialization, despite the regulatory challenge of showing both contraceptive and anti-viral efficacies.

### Speed, quality, and quantity: GMP manufacturing of HCA in *Nicotiana* for phase 1 trials

The speed to producing gram quantities of antibodies in 14 days in *Nicotiana* has been previously acknowledged [39]. However, the speed to manufacture antibodies suitable in quality (GMP) and quantity (>50 g) for phase 1 clinical trials in 8 weeks is underappreciated.

Nicotiana manufacturing technology employs a transient expression system launched by infiltration of plants with *Agrobacterium* strains carrying plasmids with plant viral genes encoding polymerases and transport functions. The technology and its applications have been described [16]; the nucleotide sequence of the variable region of H6-3C4 was inserted into vectors containing human antibody scaffolds. This system has proven versatile with demonstrated expression of numerous heterologous proteins, including cytokines, interferon, bacterial and viral antigens, growth hormone, vaccine antigens, single chain antibodies, and monoclonal antibodies (mAbs) at levels of 100 mg to 1 g of total soluble protein per kilogram of fresh biomass.

A recent GMP preclinical/clinical manufacturing campaign produced 78 g of HCA drug substance; the *N*-glycans on the Fc region are a relatively homogenous 85% GnGn. The vaginal film drug product (DP), ZB-06, is manufactured to contain 10 mg of HCA in a 200 mg polyvinyl alcohol based film containing maltitol, histidine, and polysorbate 20. The individual films are sealed in foil packets. Over 800 films of ZB-06 DP were produced for stability studies, preclinical and clinical trials. Quality assays (identity, purity, potency, and safety) for stability at 2–8 and 22–28 °C meet current target specifications. IND-enabling studies (tissue cross-reactivity, rat toxicology, rabbit vaginal irritation) are currently being conducted.

### Scale considerations for commercial manufacturing of HCA antibodies

A target for the cost of antibodies for global health products, such as MPTs, is ~$10/g [7], but the cost of goods (COGS) for most manufacturing platforms is currently at least 6- to 10-fold higher than this target. However, capacity limitations may be a greater challenge. Assuming a 10 mg dose of a human contraceptive antibody (HCA), 100 doses per person per year, and one million users/year, one metric ton is required. For perspective, an estimated 15 metric tons of antibodies (all mammalian-based) were produced in 2015, including Fc-fusion protein and antibody fragments [40].

Because of a reliance on relatively expensive equipment, an economy of scale may not be realized by mammalian cell-based production technology. This realization has fueled the development of numerous transgenic technologies during the last two decades that is intended to significantly reduce process complexity and lower production costs, and dramatically increase scale.

At industrial biotechnology companies, full-scale industrial manufacturing of proteins at quantities of 10–50 metric tons per month is possible within weeks of the creation of a final protein-based molecule, and prices are less than $1 per gram-active protein [41]. These proteins are typically enzymes for industrial purposes (e.g., detergents) but may be adaptable to production of pharmaceutical-grade antibodies, especially for topical use. Some yeast (e.g., *Pichia pastoris*) and fungus (e.g., *Trichoderma reesei*) have been evaluated for production of antibodies [42, 43] that could address the metric ton challenge for antibodies. In addition to a quality expression host, the perspective of regulatory agencies on utilization of existing infrastructure (e.g., 300,000 L fermenters) for manufacturing topical contraceptive antibodies may be crucial in avoiding enormous capital costs.

## Next generation monoclonal antibodies

### Improving potency through antibody engineering

Antibody-induced agglutination is particularly effective when trying to block the transmucosal penetration of sperm, due to the very high concentration of sperm in semen and their vigorously motile motion, both of which increase the collision frequency between sperm. Indeed, agglutinating antisperm antibodies administered to rabbit CVM effectively agglutinated sperm in vivo and reduced fertility to almost 0% in a dose-dependent manner [21].

Polyvalent immunoglobulins such as sIgA and IgM, with 4 and 10 Fab domains per molecule, respectively, are considerably more potent than IgG (with 2 Fab domains) at inducing agglutination. Increased agglutination potency can translate to both quicker and more complete agglutination of sperm in the semen, and also less amount of total antibody needed to ensure complete agglutination of all progressively motile sperm. However, compared to IgG, sIgA and IgM are considerably more difficult to produce and purify, and are substantially less stable. These shortcomings have thus far limited the development of antibodies for clinical applications to almost exclusively IgG or IgG-derivatives.

**Figure 2:**
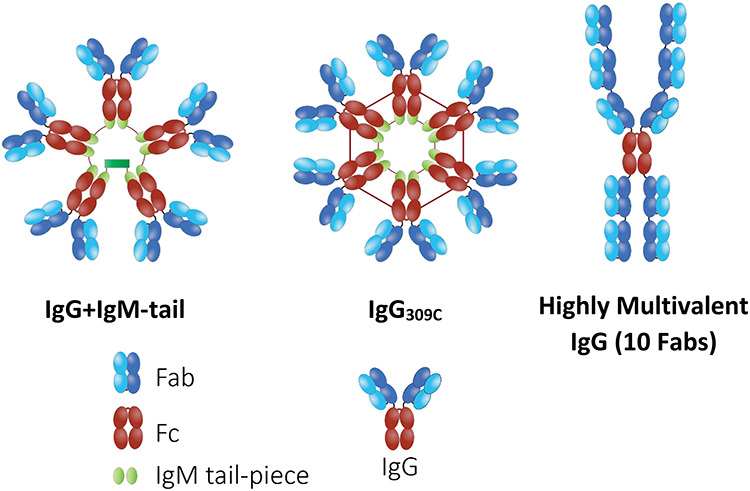
Schematic of highly multivalent HCA IgG.

Instead of relying on naturally occurring polyvalent antibody formats, advances in biotechnology over the past decade have illustrated the possibility of engineering IgG-like molecules with higher Fab valency, based on inducing self-assembly of modified IgG molecules. For instance, by modifying specific amino acids in the Fc-domain, it is possible to create specific noncovalent interactions between Fc segments of IgGs that result in the formation of ordered IgG hexamers (Figure 2) [44]. Alternatively, taking advantage of disulfide bonds between cysteines located in the secretory tailpieces of the heavy chains of IgM that is responsible for IgM’s pentameric assembly, investigators have explored incorporating the IgM tail piece into the C-terminus of IgG to create IgG pentamers (Figure 2) [45, 46].

More recently, a different approach to increasing IgG Fab valency is being explored; this strategy is based on directly encoding additional Fab domains on both the N- and/or C-terminus of the IgG heavy chain through use of flexible serine–glycine linkers (Figure 2). Since these highly multivalent IgG (HM-IgG) are monospecific, there are no issues with correct pairing of heavy and light chains of the Fab domain, a notorious challenge with other multivalent antibodies that are bispecific or trispecific. By possessing whole Fab domains that preserve the CH_1_/C_L_ interface, the HM-IgGs are more likely to possess stability, affinity, and expression profiles comparable to their parent IgG than common IgG-derivatives based on combining multiple single chain variable fragments (scFv) together. Sperm-binding HM-IgGs exhibited 4- to 16-fold greater sperm-agglutination potency than their parent IgG in vitro and in vivo (unpublished findings).

There are a number of important differences between HM-IgGs vs. multimeric IgGs. First, HM-IgGs only require the naturally high fidelity pairing of heavy chains, and thus the base molecule already possesses the increased Fab valency. In contrast, methods that require self-assembly of modified IgGs typically possess both monomeric IgGs together with one or more species of multimers (hexamers, trimers, and dimers) in the same pool, which in turn require additional purification, and are possibly less stable. Second, HM-IgGs possess only a single Fc domain per molecule, whereas the multimeric IgGs possess multiple Fcs. Depending on the application of interest, the presence of multiple Fc domains could either be an advantage (conferring enhanced effector functions) or be a disadvantage (increased immunogenicity or inflammatory response). Despite these differences, the presence of IgG-Fc with both classes of multivalent molecules ensures facile bioprocessing using conventional protein A/G chromatography, and also increased stability due to the large amount of IgG incorporated. This makes such engineered multivalent/multimeric IgGs a promising alternative to sIgA/IgM in developing more potent contraceptive immunoglobulins for vaginal passive immunocontraception.

## Delivery of contraceptive mAbs to the genital tract

Monoclonal antibody (mAb)-based MPT directed against sperm or pathogens must be present at sufficient quantities in the female reproductive tract to provide contraception and disease prevention. Vaginal films and intravaginal rings (IVR), both of which have long been used successfully to deliver various contraceptive agents, show promise for vaginal delivery of mAbs. Alternatively, antibodies can be delivered to mucosal surfaces by the systemic circulation after intravenous, intramuscular, or subcutaneous injection.

### Topical delivery

Products intended for prevention must provide a high level of safety, since they are intended for use by healthy people rather than by people suffering from established disease. To this end, the topical route of delivery may reduce safety concerns and side effects, since it avoids exposure to the full diversity of tissues and body systems exposed with systemically distributed drugs, particularly for biologics that are too large to readily enter the systemic circulation. For full realization of the desired level of safety, the topical agent must also be locally non-irritating and non-inflammatory. As normal constituents of the genital tract secretions, antibodies promise to fulfill these requirements, and several anti-HIV and an anti-HSV mAbs have been proven to have low local toxicity in Phase 1 studies [8, 47], and to have undetectable systemic absorption [8, 47].

### Vaginal film

Polymer-based thin film is an established and well accepted format for delivery of vaginal spermicide [48]. Acceptability of the film format has been high [49], likely due to the following advantages: (1) vaginal films produce less discharge than vaginal gel products [50] due to their low volume and osmotic activity. (2) Films are compact, portable, discrete, and are inserted digitally, requiring no applicator to clean, store, or dispose of. (3) Film is an “on-demand” product format, i.e., it is used only when needed, and provides rapid onset of protection. These properties may be valued by those who have infrequent intercourse, are between partners, are between contraceptive methods, or need protection on short notice.

**Figure 3:**
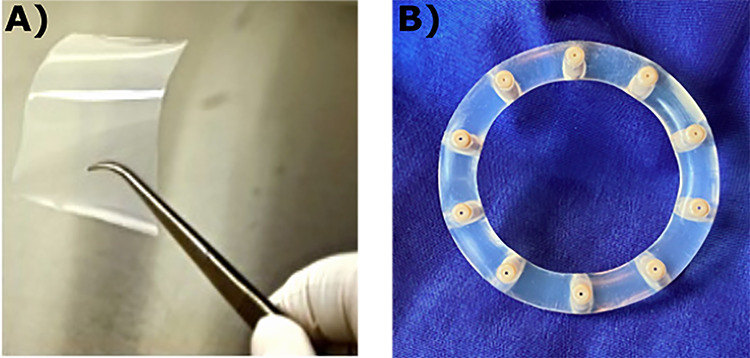
(A) MB66 film containing 10 mg of VRC01 anti-HIV bNAb, and 10 mg of HSV8 anti-HSV antibody; (B) IVR bearing antibody-loaded capsules for contraception or MPT protection.

As a result, considerable development has been undertaken to deliver small molecule anti-retroviral drugs in vaginal films for HIV prevention, with encouraging results in Phase 1 safety [50–53] and acceptability [49] studies. Vaginal film is particularly suitable for topical delivery of mAbs, since as a dry product format, it can provide long-term thermal stability for biologics [54]. A mAb-based MPT film product, MB66 film (Figure 3A), containing both an anti-HIV (VRC01) and anti-HSV (HSV8) antibodies, has recently completed Phase 1 safety and pharmacokinetic testing [8]. The film was well tolerated and acceptable, and intravaginal antibody levels at 1 and 4 h after film placement were well above the inhibitory levels for both HIV and HSV, and cervicovaginal lavage fluid at 24 h showed significant neutralization of HSV and both laboratory adapted and primary isolates of HIV [8].

### mAb intravaginal rings

Notwithstanding the benefits of the on-demand pre-coital vaginal film format for contraception and disease prevention, the typical-use effectiveness of such products is reduced by the need to apply the film in advance of each intercourse. As an alternative format, the mAb-releasing IVR is being designed to provide protection over an entire menstrual cycle with a single insertion, and does not require timed pre-coital action. Thus, the IVR format has the potential for greater convenience, higher adherence, and therefore greater effectiveness than pre-coital formats.

The IVR is well established for vaginal delivery of hormonal contraception [55] and treatment for vaginal atrophy [56]. IVR technology been recently been extended beyond its original applicability to small molecule drugs, to the delivery of large biomolecules such as mAbs [57, 58]. Sustained delivery of the anti-HIV broadly neutralizing antibody (bNAb) VRC01 has been demonstrated in macaques for up to 28 days. Steady state levels of ~100 μg/ml were achieved in vaginal fluids [58]. With its multiple, independent, antibody-containing “pods,” this ring is well suited for the development of MPT products to provide both contraception and protection against HIV/STIs. Newer IVR designs intended to simplify manufacturing and lower costs are under development (Figure 3B, Mucommune, LLC).

### Systemic delivery of antibody MPTs

Judging from the success of injectable long-lasting progestational agents for contraception [55], the systemic route of administration may prove to be an attractive mAb product format for both HIV prevention and contraception. Intravenous injection of anti-HIV bNAbs has repeatedly demonstrated protection against both rectal and vaginal challenge in macaque/SHIV models [59–63] and in humanized mouse/HIV models [64, 65]. Newer, more highly potent and broad spectrum bNAbs [66, 67] have demonstrated effective protection with sufficiently low doses to be compatible with intermittent subcutaneous injection [60, 62, 68], and antibodies with half-life-extension mutations show promise to provide protective levels for three or more months between injections [69]. Human safety trials have shown intravenous or subcutaneous administration of multiple bNAbs to be well tolerated (VRC01, VRC01LS, VRC07523LS, 3BNC117, and 10-1074) [68–72].

The systemic route may also be feasible for administration of antisperm contraceptive antibodies for extended protection. Direct measurement of the achievable levels of various forms of antisperm antibodies in the vagina and upper tract will be needed to determine the feasibility of the systemic route for mAb-based contraception.

## DNA and RNA technologies for contraception

One of the challenges with passive immunocontraception is delivery of the antibody, ensuring that the appropriate amount is delivered to the right location, as well as durability and efficacy. Novel approaches for delivering antibodies is through their expression from viruses such as adeno-associated virus (AAV) [73–75], plasmid DNA [76–79] or synthetic messenger RNA (mRNA) [78]. In this next section, we will explore these methods in more detail.

### Adeno-associated virus for intravaginal expression of an immunocontraceptive antibody

A number of viral or virus-derived methods of DNA delivery are under development [80]. One such approach for delivery of non-replicating, episomal DNA is the recombinant AAV (rAAV) [81]. AAVs are nonpathogenic, naturally replication-deficient parvoviruses which vary in serotype (AAV 1, 2, 4, 5, etc.). They are capable of transducing a variety of tissues and cell types with the potential of directing long-term expression from months to years since the vector persists predominantly in episomal form, meaning within the nucleus, but outside of the endogenous chromatin [82]. This itself may or may not be beneficial; for antibodies against HIV, where long-term expression would be beneficial for preventing infection, this would be a very useful approach, but for contraception, the effect could be permanent, depending on the cell types being transfected. The upper layer of the cervicovaginal mucosa continuously sheds, in contrast, to the basal layer of the mucosa including the epithelial stem cells, which is a replenishing source of squamous epithelial cells. If only the upper layer cervicovaginal mucosal cells were transfected, expression would be transient governed by cell shedding; however, if genital epithelial stem cells were transfected, this may produce long-term contraception. In an early paper by Abdel-Motal et al. [74], AAV 2 and 6 were found to be able to transduce human endocervical, ectocervical, and vaginal epithelial cells (VEC); given AAV6 was significantly better than 2, it was then tested with human primary genital-epithelial and epithelial stem cells. In both cases, AAV6 was successful at transducing those cell types. This approach, expressing the b12 minibody against HIV using AAV6, was then tested in an organotypic VEC tissue model. Significant levels (>12 μg/ml) of a b12 minibody were detected in the secretions after 5 days, and these tissues were resistant to infection. More recently this same group of researchers applied this approach in the macaque vaginal model [73], again using AAV6 expression of the b12 minibody. In the macaque, they found they could achieve peak values of ~800 ng/ml in the secretions, and 60–70% of peak levels was detected 79 days into their study. Even though this amount was relatively low, though likely efficacious, it demonstrated the feasibility of this approach. *Could this approach be used for contraception?* It possibly could. There are a number of factors that would influence the success of this approach. If the magnitude of expression could be increased a factor of 10, and the durability controlled, this would go a long way towards its use. Also, the ability to repeat dosing would be important; this depends heavily on the immunogenicity [83] of the AAV construct, and work is ongoing in this area. Last, there have been reports of integration of the AAV single stranded DNA into cellular genomic DNA [84] which would also be a set-back for this approach. Depending on where the integration events occur within the chromatin, this could result in gene loss of function. It would be beneficial to reduce the frequency of integration events, especially when AAV is used in relatively healthy individuals such as contraceptive users.

### Plasmid DNA

Interestingly, there have been very few attempts at using plasmid DNA intravaginally. Most of the researchers that did attempt this were using plasmid DNA as a part of DNA-based vaccine approaches. Kanazawa et al. [76] utilized pDNA expressing chicken ovalbumin (OVA) and delivered the plasmid, with and without cell permeating peptides, intravaginally via micropipette. Expression of OVA was not examined, but anti-OVA antibody and IFNγ production were measured. Both IgG and IgA as well as IFNγ were produced in an OVA specific manner demonstrating that OVA had been expressed. This work was subsequently repeated [77] using a needle-free injector in rabbits, where delivery was directly applied to the vaginal mucosa. In this study, luciferase expression was demonstrated, as well as the immune response to OVA. Though difficult to compare luciferase expression values across experiments, the quantification of the luciferase signals appeared low. In more recent work [85], a polyethylenimine-based suppository was developed to deliver DNA to the vaginal mucosa of mice; both fluorescent proteins and transcription activator-like effector nucleases were successfully delivered in this manner, with the ultimate goal of curing HPV infections via gene editing. Overall this method appeared to work well, but the extent of expression and durability were not clear. Multiple dosing regimens were utilized, from every other day, to once a week and variations on that theme. Unfortunately, the data were not displayed such that it was easy to determine how this affected protein production. Overall, there are few researchers that have attempted this approach, though the published data appear promising for this approach to be used more widely.

### Synthetic messenger RNA

In the last 10 years, there has been an explosion of research regarding the use of synthetic mRNA both for vaccine and therapeutic applications. This is mostly due to the inherent improved safety profile of mRNA over other platforms, which includes transient expression, low probability of integration, and mitigation strategies for innate immune activation [86]. Given these characteristics, mRNA should be an ideal vector for intravaginal expression of contraceptives and therapeutic proteins. To date, there have only been two publications regarding their use intravaginally, but likely there will be more to come.

The first publication in 2015 [78], described the use of microsprayer (Penn Century)-delivered mRNA and pDNA intravaginally in pigs. Here, lipofectamine, liposomes, cationic cyclodextrin, and branched PEI were used in conjunction with a microsprayer to deliver mRNA and pDNA intravaginally. The microsprayer is a small atomizer that literally allows for the atomization of drugs and the “spraying” of the drugs on the tissue surface. Characterizations were made in vitro with all of the carriers, and in vivo in a pig, where only the lipofectamine and cationic cyclodextrin were used. In the pig model, luciferase expression was examined in the female reproductive tract at 24 h via IVIS imaging. Only the pDNA/lipofectamine produced significant signals. One possible reason for the poor results from the mRNA may have been due to poor 5′ capping of the mRNA resulting in a lack of cap-dependent translation, or activation of protein kinase R (PKR) which results in reduction of eIF2a, which is critical for translation [87]. This would result in lower expression. PKR activation can be greatly mitigated by the inclusion of modified nucleotides or sequence engineering [88].

**Figure 4:**
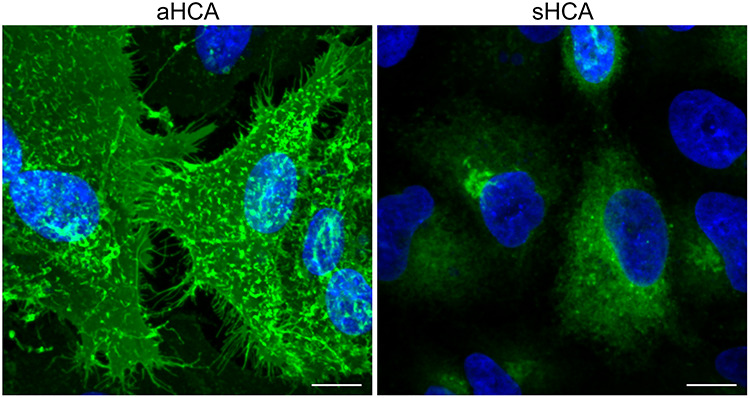
Expression of HCA in epithelial cells using synthetic mRNA. Expression of HCA (green) is demonstrated for both a GPI-anchored (aHCA) and secreted version (sHCA) of the antibody in epithelial cells; the nuclei are represented in blue. In the panel showing the anchored version of the antibody, the antibody can be seen localized to the cell membrane, which acts as a controlled release mechanism, while in the secreted case, it is observed in the ER and golgi, as it makes its way to the cell membrane and is then released into the cell media.

In a more recent publication [89], synthetic mRNA was again utilized, but to express PGT121, is a well-established HIV neutralizing antibody [90]. In this case the mRNA was modified with N-1-methylpseudouridine, which greatly mitigates innate immune responses [91] and was sprayed directly on the cervical and vaginal mucosa using a Teleflex off-the-shelf atomizer in water; no nanoparticle was used in this work. Here, significant expression of the antibody was achieved, and tissue distribution was assessed through the inclusion of nanoluciferase in the light chains of the antibody. Nanoluciferase is a relatively small (~19kD) luminescent protein that allows for protein detection and visualization in cells and tissues. Peak levels of expression were on the order of 100 μg/ml in secretions; ~10 μg/ml of antibody was detected 28 days post-delivery. The longevity of the approach was facilitated by the inclusion of a glycosylphosphatidylinositol (GPI)-anchor into the heavy chain of the antibody, which acts like a control release mechanism. GPI anchors facilitate the trafficking of the antibodies to the plasma membrane and are typically only cleaved by specific enzymes. Using the Teleflex atomizer, the tissue was penetrated deeply (~1 mm), also facilitating durability. Penetration of the tissue was not accompanied by tissue damage and therefore is unlikely to be painful. This approach has demonstrated the ability to produce significant levels of antibody, which should facilitate its use for contraception. To that aim, the Santangelo lab recently made HCA-encoding mRNA which was successfully expressed in epithelial cells (Figure 4), both with and without a GPI anchor.

Overall, there are a number of promising nucleic acid-based approaches for contraception. Only over time will it be clear which approaches will translate into human use.

## Conclusions

Several important steps have been taken in the development of an antibody-based MPT product to protect women against sexually transmitted infections and unintended pregnancies. Our first vaginal film product, MB66, containing antibodies directed against HIV-1 and HSV-1 and 2, was rapidly produced to GMP standards in an efficient *Nicotiana* platform, and used in two Phase 1 clinical trials that demonstrated safety and ex vivo efficacy after single and multiple dose applications in women. An antisperm monoclonal antibody “the HCA” has been engineered and produced in *Nicotiana* in preparation for a “proof of concept” Phase 1 clinical trial that will include safety endpoints and a post coital test that will assess the number of progressively motile sperm in cervical mucus after intercourse in tubally ligated women that use the product. At the same time our team is exploring more effective antibody designs, production and delivery methods in preparation for a second generation MPT product to address unmet needs in reproductive health.

## Conflict of interest

KJW and LZ own Mappbio, a company that intends to commercialize antibody-based products. SKL, TRM and RAC work for Mucommune, a company that is designing antibodies and intravaginal rings. The other authors have no conflicts.
